# Therapeutic effect of daphnetin on the autoimmune arthritis through demethylation of proapoptotic genes in synovial cells

**DOI:** 10.1186/s12967-014-0287-x

**Published:** 2014-10-14

**Authors:** Kuanyong Shu, Nanzhen Kuang, Zhiqin Zhang, Ziling Hu, Yujuan Zhang, Yingyuan Fu, Weiping Min

**Affiliations:** Department of Immunology, Medical College of Nanchang University; Institute of Immunology and Immunotherapy, Nanchang University and Jiangxi Academy of Medical Sciences, Nanchang, China; Department of Gynecological Oncology, Jiangxi Maternity and Child Healthcare Hospital, Nanchang, China; Reproductive Center, Jiangxi Maternal and Child Health Care Hospital, Nanchang, China; Affiliated Stomatological Hospital of Nanchang University, Nanchang, China

**Keywords:** Daphnetin, 5-aza-dc, Synovial cells, CIA rats, Demethylation, Apoptosis

## Abstract

**Background:**

We have previously reported that dephnetin is therapeutically effective in the treatment of rheumatoid arthritis (RA) in collagen-induced arthritis (CIA) rat model. However, the molecular mechanism and the effect of daphnetin on demethylating proapoptotic genes in the synovial cells remains further clarified. This study may provide a deeper insight into the medicinal application of daphnetin as a treatment for RA.

**Methods:**

(1) The proliferation inhibition of CIA rat synovial cells was determined by an MTT (3-(4,5)-dimethylthiahiazo(-z-y1)-3,5-di-phenyterazoliumromide) assay; (2) Methylation specific PCR (MSP) was used to measure the methylation of the proapoptotic genes DR3 (death receptor 3), PDCD5 (programmed cell death 5), FasL and p53; (3) Real time-PCR was performed to determine the mRNA expression of DR3, PDCD5, FasL, p53 and DNA methyltransferases (DNMTs) DNMT1, DNMT3a and DNMT3b; (4) Flow cytometry was applied to detect the protein expression of the DR3, PDCD5, FasL and p53; (5) The apoptotic rate of synovial cells was assessed by flow cytometry with Annexin V and propidium iodide (PI); (6) Scanning electron microscopy (SEM) and transmission electron microscopy (TEM) were used to observe the changes of CIA rat synovial cell structure.

**Results:**

(1) In the range of 1.25 μg/mL to 40 μg/mL, daphnetin and 5-aza-dc had a dose-dependent and time-dependent degree of inhibition to the CIA rat synovial cells. (2) Daphnetin and 5-aza-dc had a demethylating role on the proapoptotic genes DR3, PDCD5, FasL and p53 of CIA rat synovial cells. (3) Daphnetin and 5-aza-dc decreased the gene expression of methyltransferases DNMT1, DNMT3a and DNMT3b, and increased expression of proapoptotic genes DR3, PDCD5, FasL and p53, which translated into an increased protein expression of DR3, PDCD5, FasL and p53. (4) Daphnetin and 5-aza-dc changed the structure of CIA rat synovial cells to show apoptotic changes and increased the rate of apoptosis.

**Conclusions:**

Daphnetin can reduce the expression of DNMT1, DNMT3a and DNMT3b, which could result in the proapoptotic genes DR3, PDCD5, FasL and p53 being demethylated. Therefore, daphnetin can increase proapoptotic gene and protein expression resulting in structural apoptotic changes and an increase in early and late CIA rat synovial cell apoptosis.

## Introduction

Rheumatoid arthritis (RA) is a common systemic autoimmune disease which is characterized by chronic inflammation of the joints, hyperplasia of synovial cells and progressive destruction of cartilage and bone, leading to joint deformity, dysfunction, and varying degrees of disability. According to the statistics, RA is affecting approximately 1% of the population [1]. Presently, drugs used in the treatment of RA are mainly derived from Western and Chinese medicine. However, since traditional Chinese medicine usually reduces the side effects of Western medicine and reduce disease recurrence rate, it is more promising to look for an effective drug derived from traditional Chinese medicine or active components of Chinese medicine for RA treatment and to discuss their mechanisms of resistance. Daphnetin (7, 8-dihydroxycoumarin) extracted from *Daphne odora* Var. *Marginata* (*D. marginata*) has been used to treat various autoimmune diseases including rheumatoid arthritis [2]. Our preliminary studies also found daphnetin could augment T_h_17 cells and inhibit T_reg_ cells to reduce the swelling and inflammation in the feet of CIA rats, which finally relieved articular cartilage degeneration [3,4].

The pathogenesis of RA is not completely understood, yet hyperplasia of the synovial cell is considered to be one of the main factors towards the development of RA [5]. Apoptosis plays a pivotal role in tissue homoeostasis both under physiological and pathological conditions, and some characteristic changes in the composition and structure of the inflamed synovial membrane in RA are linked to a lowered apoptotic response of synovial cells [6]. RA synovial fibroblasts (RASFs) are versatile cells with the potential to activate an array of genes that are able to initiate and propagate inflammation in RA-affected joints [7]. Studies found that rat joint synovial cell apoptosis is insufficient in type II collagen induced arthritis (CIA), internationally recognized as the most commonly used animal model to research rheumatoid arthritis. However, there are no relevant reports so far about RASFs’ ability to reduce apoptosis as a treatment for RA.

DNA methylation is the most studied and the most important apparent genetic modification form. This mainly occurs by the covalent bonding of a methyl group on the fifth carbon atom in cytosine residues, thus cytosine is modified to become 5-methyl-cytosine (5 mC). Recent research shows that DNA methylation can be affected by environmental factors, including prenatal smoking, drinking and environmental pollutants [8-11]. RA pathogenesis is thought to be due to the combination of genetic susceptibility, immune regulation disorders and environmental factors [12-14]. As well, other studies have reported mammalian gene function may be affected by apparent genetic modification without changes to the DNA sequence, such is the case with methylating DNA [15]. The complex etiopathology of autoimmune rheumatic disorders has been attributed to crosstalk between genetic predisposition and environmental factors, but recent advances in understanding epigenetic mechanisms, especially DNA methylation in the context of RA, may provide an important link regarding the pathogenesis of RA and may inspire development of new targeted therapeutic approaches [16]. High DNA methylation can inactivate gene transcription, leading to the development of disease. Luckily, DNA methylation can be reversed through demethylating drugs such as the DNA methyltransferase inhibitor 5-aza-dc. In 2011, Francesca Meda [17] proposed that epigenetics may be better able to explain autoimmune disease, but research for epigenetic mechanisms in abnormal gene expression remain confined to a small number of laboratories. One such laboratory, run by Takami N, found that the RA proapoptotic gene DR3 shows an abnormally high methylation status, which may be the cause for the lowered apoptotic response of synovial cells [18]. Therefore, if we can prove there is a relationship between the expression of RA proapoptotic genes, abnormal apoptosis and epigenetics, as well as understand the demethylating molecular mechanism and effects of daphnetin on the proapoptotic genes in the CIA rat synovial cells, we may be provided with a new method and treatment for RA.

## Materials and methods

### Cell culture and treatment

The CIA rat synovial cells were purchased from the American Type Culture Collection (ATCC, USA). They were maintained in Dulbecco’s Modified Eagle Medium (DMEM) (Gibco, USA) with 25 mM glucose, supplemented with 10% fetal bovine serum (FBS), nonessential aminoacids, 100 IU/mL penicillin, 100 mg/mL streptomycin and 2 mM glutamine. Cells were routinely cultured in 75 cm^2^ tissue culture flasks and incubated in a humidified atmosphere of 5% CO2/95% air at 37°C. CIA rat synovial cells were treated with daphnetin (40 μg/mL), 5-aza-dc (20 μM) or with the combination of them suspended in complete DMEM medium over a time course of 72 h.

### Cell viability assay

Determination of cell growth inhibition was performed using a MTT assay. To investigate the effect of daphnetin and 5-aza-dc on CIA rat synovial cell viability and proliferation, 5000 cells per well were grown in a 96-well microtiter plate in complete DMEM medium as control or supplemented with Daphnetin(1.25 ~ 40 μg/mL) or 5-aza-dc (1.25 ~ 20 μM) for 36, 48, 60 and 72 h. The cells were then incubated with 20 μL MTT solution (5 mg/mL) for 4 h at 37°C. After this incubation period, purple formazan salt crystals were formed. After careful removal of the medium, 0.1 mL DMSO was added to each well. The absorbance was recorded at a wavelength of 572 nm using a Universal Microplate Reader (EL800, BIO-TEK Instruments Inc.). The effect of Daphnetin and 5-aza-dc on cell growth inhibition was assessed as a reduction in percent cell viability, where vehicle-treated cells were taken as 100% viable.

### Methylation specific PCR

Genomic DNA was collected from cells by the TIANamp Genomic DNA Kit (Tiangen biotech co., LTD., Beijing, China). DNA was quantified with the use of an ultraviolet spectrophotometer and analyzed by 1% agarose gel electrophoresis after bisulphite modification according to the EZ DNA Methylation-Gold™ Kit (ZYMO RESEARCH, American). Methylation and demethylation primers were designed with the online Methprimer software (Table 1) and then synthesized by the Shanghai ShengGong biological engineering company. MSP was performed in the ABI 7500 sequence detection system (Applied Biosystems). Each MSP was conducted by adding 5 μL of RNase-free ddH2O, 0.5 μL of forward and 0.5 μL of reverse primer of methylation or demethylation (10 μM), 10 μL 2 × SuperReal PreMix, 2 μL 50 × ROX Reference Dye, 2 μL cDNA templates in a total volume of 20 μL. The MSP protocol consisted of 1 cycle at 95°C for 10 min, 40 cycles at 95°C for 10 s, 60°C for 20 s and 72°C for 31 s. MSP products underwent 2% agarose gel electrophoresis (constant voltage 120 v, 40 min) and an image was taken using a gel image analysis system (BIO-RAD, Germany) under ultraviolet light.

**Table 1:** **Primers of DR3, PDCD5, p53 and FasL for MSP**

**Target gene**	**Sense sequence(5′-3′)**	**Antisense sequence(5′-3′)**
DR3(M)	GAGGGTTTAGGGGAGTTTTTTAAC	AAAACCTATACTTCCTCTCAACGAA
DR3(U)	GGGTTTAGGGGAGTTTTTTAATGT	AAAACCTATACTTCCTCTCAACAAA
PDCD5(M)	AAATTGTTAAAGATATTTAATGCGT	TAAAATCTACCAATCCAAAAACGAC
PDCD5(U)	AAATTGTTAAAGATATTTAATGTGT	TAAAATCTACCAATCCAAAAACAAC
p53(M)	GCGAAGGTAGGAGTTTTAAGAGTAC	CACTATTTACGAAACAACCCGAC
p53(U)	TGGTGAAGGTAGGAGTTTTAAGAGTAT	AACACTATTTACAAAACAACCCAAC
FasL(M)	ATTTAGGAGAATTAGTAGTTGGGGC	TAAAATAAACACCTTACCTCTCTCG
FasL(U)	TTTAGGAGAATTAGTAGTTGGGGTG	CTAAAATAAACACCTTACCTCTCTCAC

### RNA Extraction and gene expression analysis

The expression of DR3, PDCD5, FasL, p53 and methyltransferases DNMT1, DNMT3a and DNMT3b in CIA rat synovial cells was measured by real-time PCR. First, total RNA was isolated from the cultured cells using the RNeasy Micro Kit (Tiangen biotech co., LTD., Beijing, China) according to the manufacturer’s procedure. One microliter of total RNA was reverse-transcribed using a 1st Strand cDNA Synthesis Kit (Takara Bio, Shiga, Japan). Gene-specific primers were used for quantitative real-time PCR; the primers are shown in Table 2. Real-time PCR was performed in the ABI 7500 sequence detection system (Applied Biosystems) with a reaction mixture that consisted of SYBR Green 2 × PCR Master Mix (Tiangen biotech co., LTD., Beijing, China), cDNA template, forward primer and reverse primer. Each real-time PCR was composed by adding 16 μL of H_2_O, 1 μL of forward and 1 μL of reverse primer (10 μM), 25 μL 2 × SuperReal PreMix, 5 μL 50 × ROX Reference Dye, 2 μL cDNA templates in a total volume of 50 μL. The real-time PCR protocol consisted of 1 cycle at 95°C for 15 min, 40 cycles at 95°C for 10 s, 60°C for 20 s and 72°C for 31 s. Data of real time-PCR were analyzed using the ABI 7500 sequence detection system software and the amounts of DR3, PDCD5, FasL, p53 and methyltransferases DNMT1, DNMT3a and DNMT3b mRNA were normalized to β-actin using the CT method.

**Table 2:** **Primers of DR3, PDCD5, p53 and FasL for real-time PCR**

**Target gene**	**Locus**	**Sense sequence(5′-3′)**	**Antisense sequence(5′-3′)**	**Product size(bp)**
DR3	NM_001137644.1	gctacgaacctacaacataccg	acagtcccaagaaggaacgag	181
PDCD5	NM_001106247.1	acgaaagcagtggagaactacc	ttcttttctgtctgttggctga	119
p53	NM_030989.3	gcccatccttaccatcatcac	cacaaacacgaacctcaaagc	80
FasL	NM_012908.1	ggtgctaatggaggagaagaag	aaatggtcagcaacggtaagat	105
DNMT3a	NM_001003958.1	gtgcttaccaatacgatgacga	atccacacactccacacaaaag	122
DNMT3b	NM_001003959.1	ggtaggagatggagatggtgaa	atactgttgctgtttcgggttc	138
DNMT1	NM_053354.3	gtgtggtgtctgtgaggtctgt	gtttcttcttcttcccttggtg	221
β-actin	NM_001009180.2	tgacaggatgcagaaggaga	tagagccaccaatccacaca	106

### Flow cytometric analysis

To detect protein expression of the DR3, PDCD5, FasL and p53, CIA synovial cells were treated with or without daphnetin (40 μg/mL) or 5-aza-dc (20 μM) for 72 h, collected by trypsinization and washed twice with PBS. After collection cells were stained with fluorescein isothiocyanate (FITC)-labeled rabbit anti-rat DR3, PDCD5, FasL and p53 (Wuhan Boster Biological Technology., LTD.). Cells were fixed and then analyzed by flow cytometry. Data acquisition and analysis were performed in a Becton Dickinson FACS Calibur flow cytometer using Cell Quest software.

### Apoptosis analysis by Annexin V/Propidium Iodide (PI) flow cytometry assay

Apoptosis of CIA rat synovial cells was examined using a FITC-labeled Annexin V/propidium iodide (PI) Apoptosis Detection Kit (KeyGen Biotechology Co. Ltd., Nanjing, China) according to the manufacture’s protocol. Cells treated with or without Daphnetin (40 μg/mL) or 5-aza-dc (20 μM) for 72 h were collected by trypsinization and washed twice with PBS. The cells were resuspended in 500 μL binding buffer. Then 5 μl Annexin-V-FITC and 5 μl PI-FITC were added and incubated in the dark for 30 min at room temperature. The labeled cells were analyzed by flow cytometry. Data acquisition and analysis were performed in a Becton Dickinson FACS Calibur flow cytometer using Cell Quest software.

### Observation of CIA rats synovial cell ultrastructure by SEM and TEM

SEM: CIA rat synovial cells were settled on Poly-l-Lysine coated coverslips (12 mm) in a 24-well plate at a density of 2 × 10^6^ cells/mL, fixed with 6.25% glutaraldehyde in 50 mM phosphate buffer, pH 7.2, for 10 minutes at room temperature, and subsequently incubated overnight at 4°C. After washing with phosphate buffer, the samples were dehydrated stepwise in acetone, critical-point dried and sputtered with platin/palladium. Cells were examined by SEM analysis, using a Zeiss DSM 962 scanning electron microscope(S-3000 N, Japan).

TEM: Cultures of CIA rat synovial cells were chemically fixed in an aqueous solution of glutaraldehyde (2.5%) in 0.1 M sodium cacodylate buffer (pH 7.4) for two days at 4°C. Decalcification was achieved by treatment with 5% nitric acid for seven days. The samples were rinsed for 24 hours, then post-fixed with a 1% osmium tetroxide solution (in 0.1 M sodium cacodylate buffer, pH 7.4) over a period of four hours at 4°C. The specimens were then washed four times in isotonic sodium cacodylate buffer (0.1 M; pH 7.4) and dehydrated through a graded series of ethanol and ethyl carbinol. Thereafter, the specimens were embedded in epoxy resin. Polymerization was performed at 60°C over 48 hours. Thin sections were stained with uranyl acetate and lead citrate and then examined by transmission electron microscopy (Hitachi H-600, Japan).

### Statistical analysis

All data represent at least three independent experiments and were expressed as mean ± standard deviation (S.D.). Parametric was evaluated by one-way ANOVA followed by LSD post hoc multiple comparison test. A T-test was used to determine the significances of differences in multiple comparisons. Values of P < 0.05 were considered statistically significant in all cases.

## Results

### Effect of daphnetin and 5-aza-dc on cell viability

**Figure 1:**
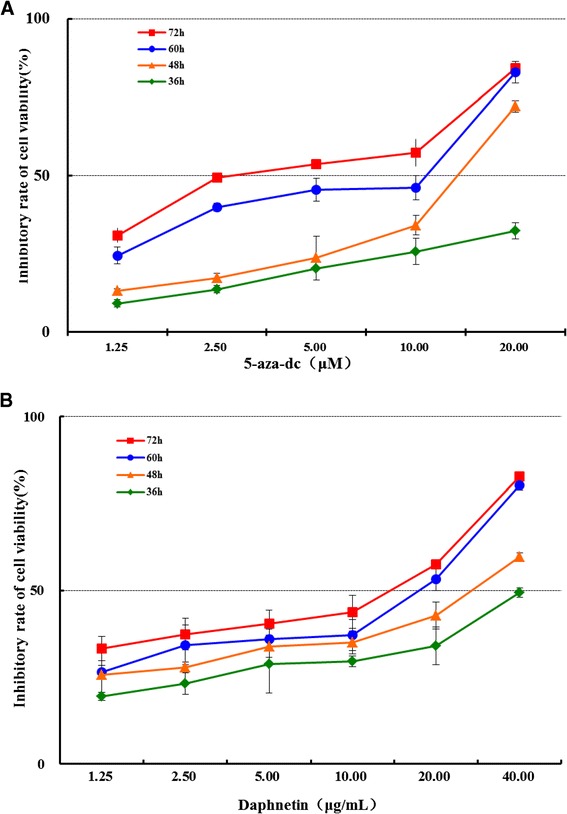
**Effect of daphnetin and 5-aza-dc on the viability of CIA rat synovial cells.** MTT assay was used to detect cell viability after treatment with varying concentrations of daphnetin **(A)** or 5-aza-dc **(B)** for 36 h, 48 h, 60 h and 72 h. The data shown are the mean from three parallel experiments.

MTT assay was used to evaluate the inhibitory effect of daphnetin and 5-aza-dc on CIA rat synovial cells. As shown in Figure 1, treatment with daphnetin and 5-aza-dc both reduced cell growth of to CIA rat synovial cells in significant dose-dependent and time-dependent fashion. The data verify that daphnetin has similar inhibitory effect, as comparing with 5-aza-dc, on the proliferation of synovial cells in vitro.

### Effect of daphnetin and 5-aza-dc on methylation of in CIA rat synovial cells

**Figure 2:**
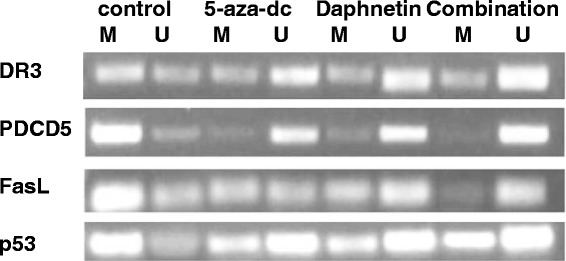
**Methylating effect of daphnetin and 5-aza-dc on DR3, PDCD5, FasL and p53 in CIA rat synovial cells.** Four experimental groups consisting of untreated cell control, or treated with 5-aza-dc at 20 μM, daphnetin at 40 μg/mL, or combination of 5-aza-dc (20 μM) and daphnetin (40 μg/mL). Cells were cultured, treated and harvested as described in Materials and Methods. Total DNA was extracted and used for MSP after bisulphite modification. M: methylated amplification products, U: hypomethylated amplification products.

Next, we detected DNA mathylation of proapoptotic genes DR3, PDCD5, FasL and p53 in the synovial cells after treatment of daphnetine and 5-aza-dc. MSP results (Figure 2) showed that methylation primer and the demethylation primer amplification specific strips of the proapoptotic genes DR3, PDCD5, FasL and p53 were all out in the untreated CIA rat synovial cells. The methylated band brightness of PDCD5, DR3, FasL and p53 was greatly reduced and the corresponding demethylated band brightness was improved after the treatment with daphnetin and 5-aza-dc. The combination group, compared to the control CIA, showed the greatest change in demethylation status. The combination group showed no methylation band of the PDCD5 gene, which indicates that PDCD5 was in a completely demethylated state. These data suggest that daphnetin possess stronger capacity than 5-aza-dc in term of inducing DNA demethylation of proapototic genes in the joints of CIA rats.

### Effect of daphnetin and 5-aza-dc on expression of DR3, PDCD5, FasL, p53 and methyltransferases DNMT1, DNMT3a and DNMT3b in CIA rat synovial cells

**Figure 3:**
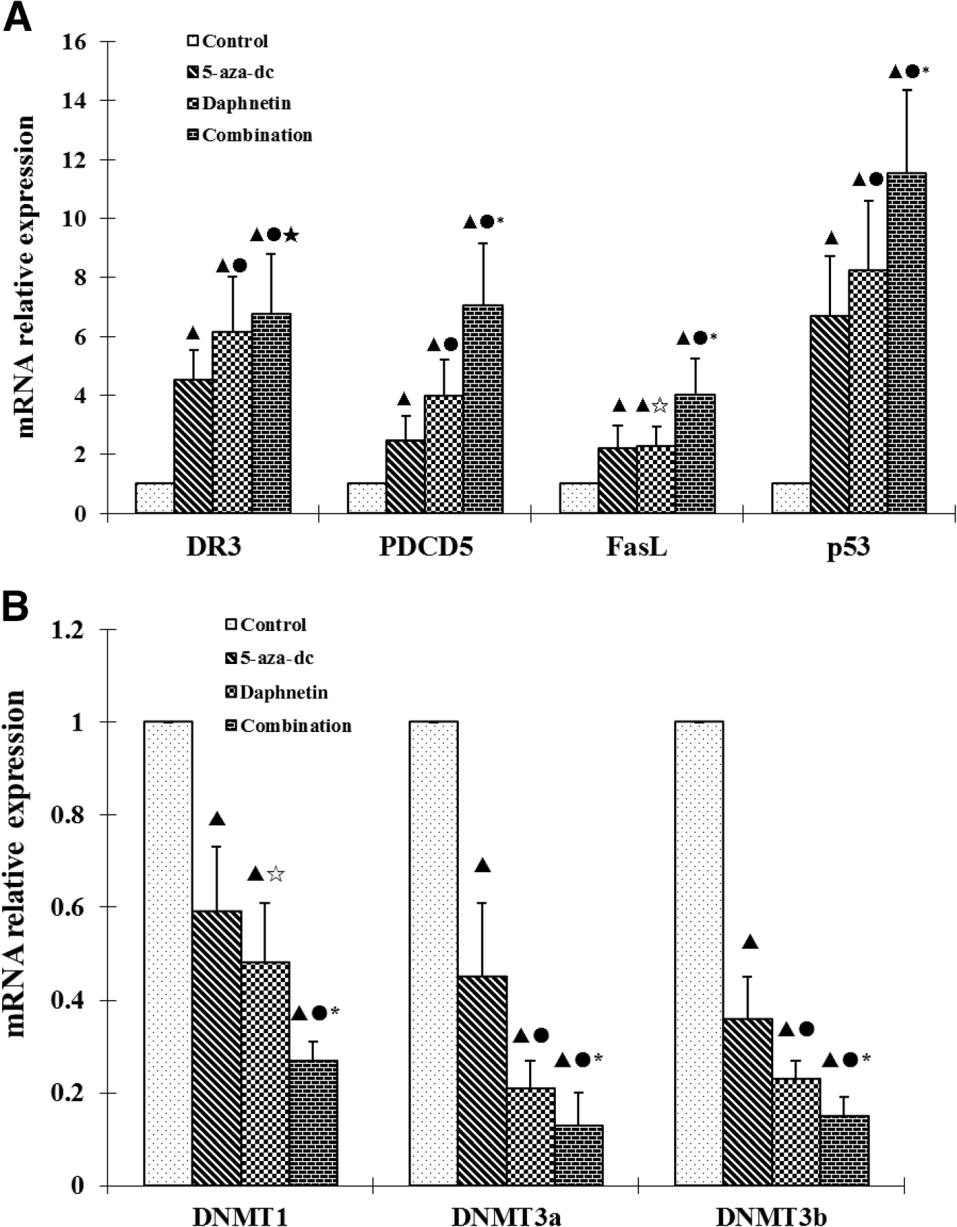
**Effect of daphnetin and 5-aza-dc on expression of DR3, PDCD5, FasL, p53 (A) and DNMT1, DNMT3a, DNMT3b (B) in CIA rat synovial cells.** Four experimental groups consisting of untreated cell control, or treated with 5-aza-dc at 20 μM, daphnetin at 40 μg/mL, or combination of 5-aza-dc (20 μM) and daphnetin (40 μg/mL). Cells were cultured, treated and harvested as described in Matreials and Methods. Total RNA was extracted and cDNA was synthesized. After reverse transcription, cDNA was used for real time-PCR. Relative quantification of gene expression was performed by the 2-∆∆Ct method. The results show the mean ± S.D. of six independent experiments. ▲P < 0.05 compared with control, ●P < 0.05 compared with 5-aza-dc, ☆P > 0.05 compared with 5-aza-dc, ★P > 0.05 compared with daphnetin, *P < 0.05 compared with daphnetin.

Real-time quantitative PCR was used to indicated the effect of daphnetin and 5-aza-dc on expression of DR3, PDCD5, FasL, p53 and methyltransferases DNMT1, DNMT3a and DNMT3b in CIA rat synovial cells. The results showed that DR3, PDCD5, FasL and p53 mRNA were expressed at significantly higher levels in daphnetin and 5-aza-dc treated cells compared to control (Figure 3A). DR3, PDCD5 and p53 mRNA were expressed at significantly higher levels in daphnetin than in 5-aza-dc treated cells. At the same time, DR3, PDCD5, FasL and p53 mRNA levels in the combined treatment were the highest of the four groups (Figure 3A). However, DNMT1, DNMT3a and DNMT3b were expressed at significantly lower levels in daphnetin and 5-aza-dc treated cells compared to control (Figure 1B). DNMT3a and DNMT3b mRNA were expressed at significantly lower levels in daphnetin than in 5-aza-dc treated cells. Lastly, DNMT1, DNMT3a and DNMT3b mRNA levels in the combination were the lowest of the four groups. These data imply that daphnetin and 5-aza-dc are capable of upregulating the expression of proapoptotic gene (ie., DR3, PDCD5, FasL and p53 mRNA) though degrading the methyltransferases DNMT1, DNMT3a and DNMT3b.

### Effect of daphnetin on protein expression of DR3, PDCD5, FasL and p53 in CIA rat synovial cells

**Figure 4:**
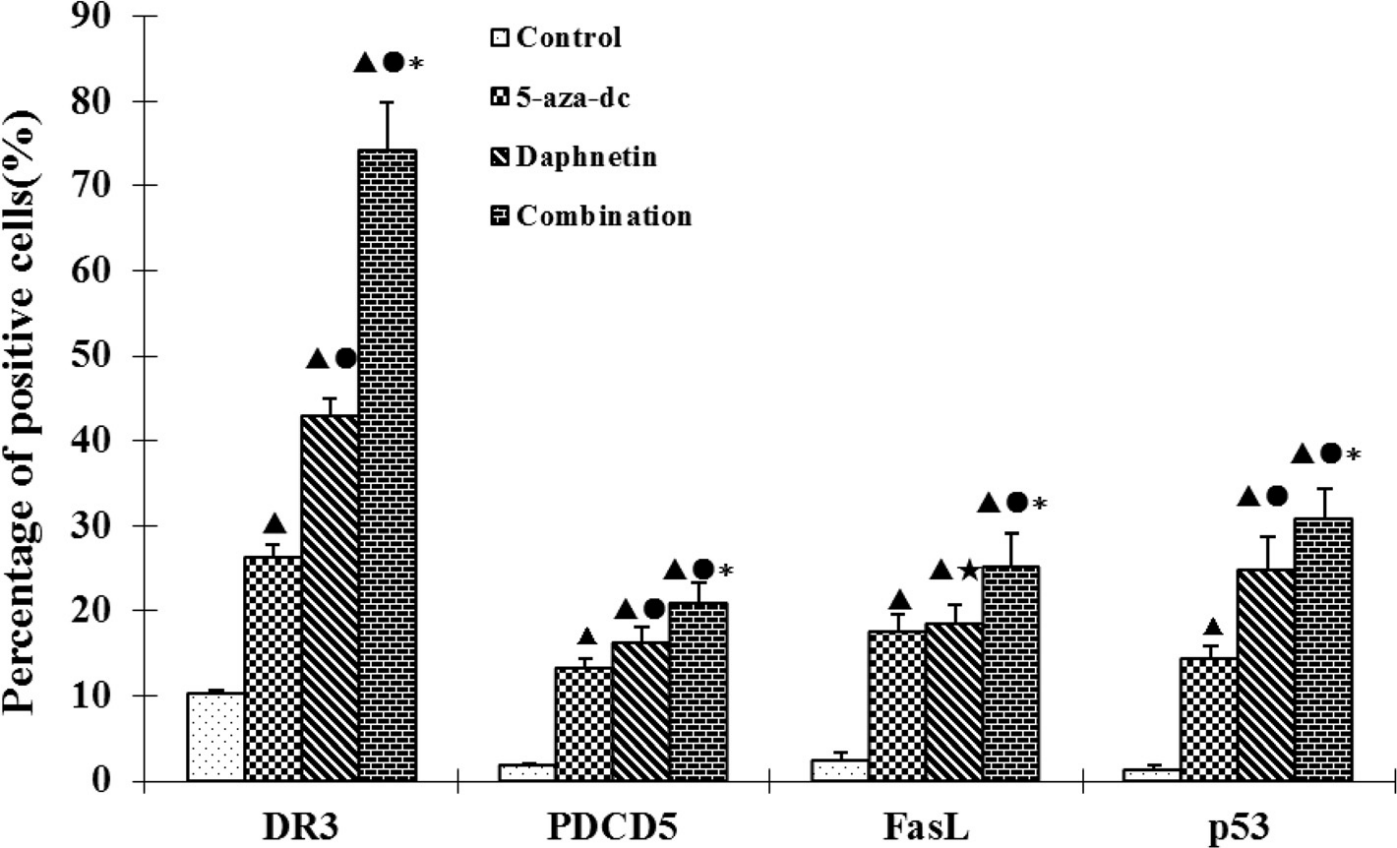
**Effect of daphnetin on DR3, PDCD5, FasL and p53 protein expression in CIA rats synovial cells.** Cells were cultured, treated and harvested as described Materials and Methods. Four experimental groups consisting of untreated cell control, 5-aza-dc at 20 μM, daphnetin at 40 μg/mL, or combination of 5-aza-dc (20 μM) and daphnetin (40 μg/mL). The results show the mean ± S.D. of six independent experiments. ▲P < 0.05 compared with control, ●P < 0.05 compared with 5-aza-dc, ★P > 0.05 compared with 5-aza-dc, *P < 0.05 compared with daphnetin.

Flow cytometry analysis was used to detect the effect of daphnetin on protein expression of DR3, PDCD5, FasL and p53 in CIA rat synovial cells showed that DR3, PDCD5, FasL and p53 proteins were expressed at significantly higher levels in daphnetin and 5-aza-dc treated cells compared to control (Figure 4). DR3, PDCD5 and p53 protein were expressed at significantly higher levels in daphnetin than in 5-aza-dc. At the same time, DR3, PDCD5, FasL and p53 protein in the combination were the highest of the four groups. The data uncover the upregulated expression of proapototic gene DR3, PDCD5, FasL and p53 in the joints of CIA mice after treatment with daphnetin and 5-aza-dc.

### Possible involvement of apoptosis in daphnetin and 5-aza-dc-induced cell death

**Figure 5:**
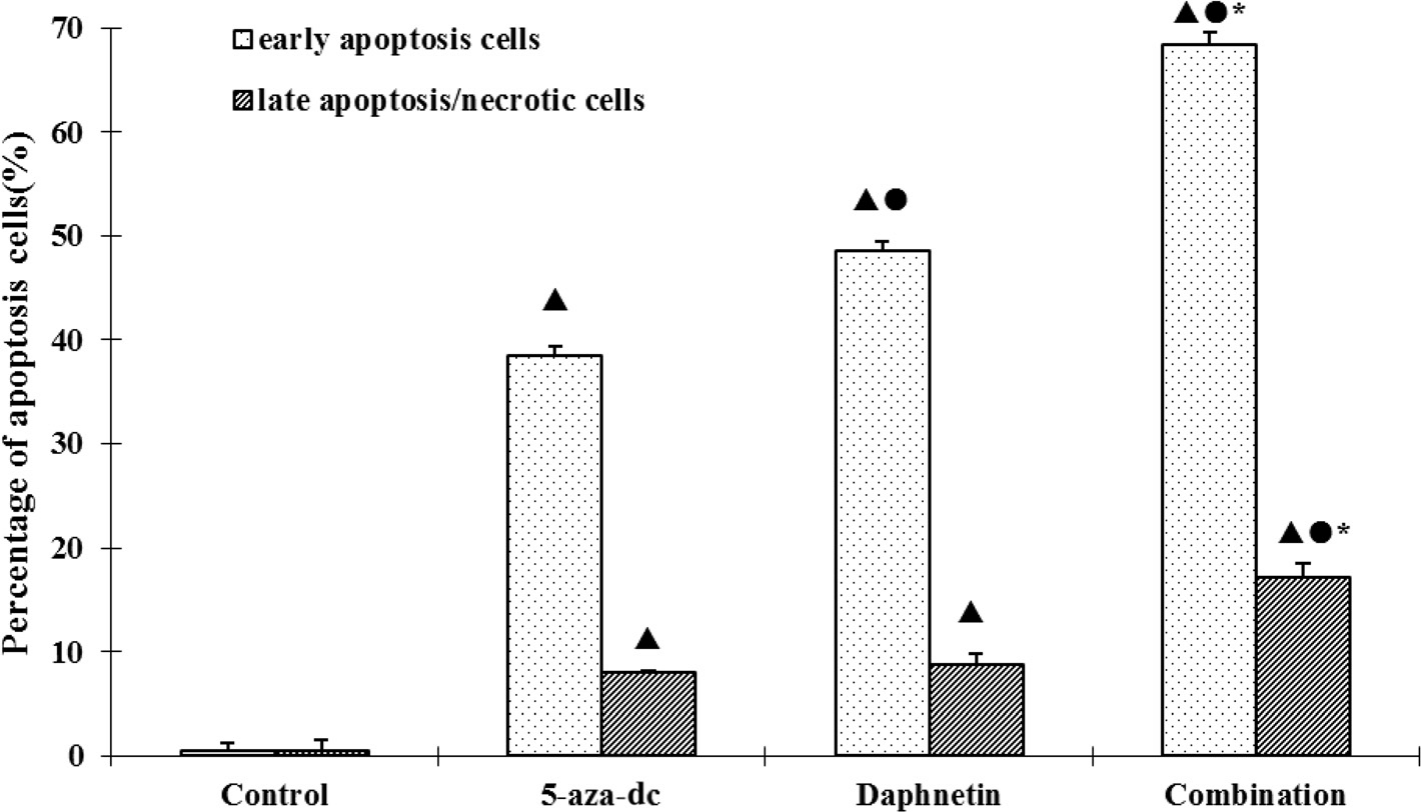
**Cell apoptosis/necrosis as measured by Annexin V/PI staining.** Four experimental groups consisting of untreated cell control, 5-aza-dc at 20 μM, daphnetin at 40 μg/mL, or combination of 5-aza-dc (20 μM) and daphnetin (40 μg/mL) for 72 h. Cells were then processed for Annexin V/PI staining and analyzed by flow cytometry. The results show the mean ± S.D. .of six independent experiments. ▲P < 0.05 compared with control, ●P < 0.05 compared with 5-aza-dc, ☆P > 0.05 compared with 5-aza-dc, *P < 0.05 compared with daphnetin.

Annexin V/PI staining was applied to detect apoptotic cells after daphnetin and 5-aza-dc treatment. Figure 5 showed that treatment with daphnetin and 5-aza-dc significantly induced apoptosis in CIA rat synovial cells. The combination induced the highest amount of necrosis. These data suggested that apoptosis is involved in suppression of synovial cell proliferation in CIA rats after treatment with daphnetin and 5-aza-dc.

### Effect of daphnetin and 5-aza-dc treatment on the structure of CIA rat synovial cells

**Figure 6:**
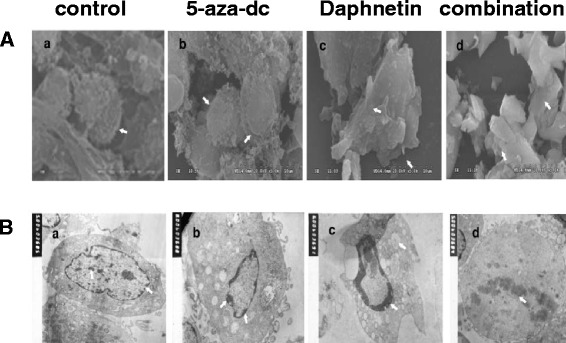
**Effect of daphnetin and 5-aza-dc on the structure of CIA rat synovial cells.** Four experimental groups consisted of untreated cell control, 5-aza-dc at 20 μM, daphnetin at 40 μg/mL, or combination of 5-aza-dc (20 μM) and daphnetin(40 μg/mL). **(A)**: SEM; **(B)**: TEM.

Scanning electron microscopy and transmission electron microscopy were used to detecte the effect of daphnetin and 5-aza-dc treatment on the structure of CIA rat synovial cells. The scanning electron microscopy showed that the CIA rat synovial cells had round or oval shape and their surface had fine microvilli hooks; the cells were slightly deformed in the 5-aza-dc group, including a reduction in the surface microvilli compared to the control group; meanwhile the cells in the daphnetin group had a serious deformation and the cell surface microvilli hooks disappeared and were replaced with small fingerlike projections; the synovial cells in the combination group displayed extreme deformation, a smooth surface and a seriously burst surface (Figure 6A).

Transmission electron microscopy showed visible surface villi, clear nuclear membrane structure and prominent nucleoli in the untreated CIA rat synovial cells (Figure 6B). The nuclear pleomorphism showed that cytoplasm organelles were well developed; the 5-aza-dc group cells displayed a reduction in size, irregular form, disappeared surface microvilli, a clear double layer, pyknotic nuclear chromatin complexed together within the cytoplasm of a few rough endoplasmic reticulum, increased number of lysosomes, developed free ribosomes and mitochondrial swelling and empty bubble degeneration, which all demonstrate apoptotic changes; the synovial cells in the daphnetin treatment group had further apoptotic developments compared with the 5-aza-dc group; the nuclear membrane obviously disappeared and apoptotic bodies formed in the synovial cells of the combination group. Taken together, the electronic microscopic scans further verify the alternations of apoptosis in the joints of CIA rats after daphnetin and 5-aza-dc treatments.

## Discussion

Rheumatoid arthritis joint synovial cells have completely transformed cell characteristics, which may be due to the inflammation environment [19]. The modification of mammalian genomic DNA includes the methylation at the 5^th^ position of the cytosine (C) residue within cytosine-guanine dinucleotides (CpGs), resulting in the formation of 5-methyl-cytosine (5mC). There are three kinds of mechanisms that can inactivate genes, and in some cases methylation is the only active mechanism. Fortunately, DNA methylation is reversible and this feature promoted the development of DNA demethylation drugs such as 5-aza-dc, a DNA methyltransferase inhibitor that can specifically inhibit the activity of methyltransferase, decrease the level of DNA methylation and inhibit the growth of cells [20]. Another study suggested that DNA hypomethylation contributes to the chronicity of RA and could be responsible for the limitation of current therapies [21]. At the same time Meinecke I discovered the role of RA synovial fibroblasts (RASFs) in the initiation and progression of joint destruction in RA [22]. Therefore, the role of the methylation status of DNA is an important factor in RA pathogenesis, however, currently there are no relevant reports about the methylation status of proapoptotic genes in CIA rat synovial cells.

Death receptor 3 (DR3) participates in the apoptosis signal path and is often the target of gene silence. Takami N found that the CpG islands in the DR3 gene promoter were specifically methylated to down-modulate the expression of DR-3 protein in rheumatoid synovial cells, which may provide resistance to apoptosis [18]. Programmed death gene 5 (PDCD5) has an effect of promoting tumor cell apoptosis and inhibiting tumor cell proliferation. A study found that PDCD5 may serve as a therapeutic target to enhance sensitivity to antirheumatic drug-induced apoptosis in RA [23]. FasL belongs to TNF/LT super family members. The combination of FasL and Fas can lead to Fas-induced cells apoptosis. Experiments have proved that Fas mRNA expression is higher in the RA synovium culture cell in vitro, suggesting that it may be in a state of activation, yet FasL mRNA expression was not found [24] and the reason behind the lack of FasL expression is not clear. p53 is classic tumor suppressor gene and plays an important role in cell proliferation, DNA repair and apoptosis. It was found that p53 in RASFs can adjust the function of T_h_1 and T_h_17 cells, and it plays an important role in the pathogenesis of RA [25]. There have many reports about the methylation status of p53 in other diseases [26-28], but there has not been any related research in RA. Our results indicated that DR3, PDCD5, FasL and p53 in CIA rat synovial cells were all in a state of hypermethylation and treatment with daphnetin, lowered the states of methylation in these genes. At the same time, the expression of DR3, PDCD5, FasL and p53 in the daphnetin group was significantly increased. Moreover, daphnetin worked better than the demethylation reagent 5-aza-dc. So we proposed that daphnetin decreases the expression of methyltransferases which results in the demethylation of proapoptotic genes such as DR3, PDCD5, FasL and p53, therefore enhancing proapoptotic gene expression and the rate of apoptosis.

The methylation of mammalian genomic DNA is catalyzed by DNA methyltransferases (DNMTs), which play a special role in the initiation of chromatin remodeling and gene expression regulation. The mammalian DNMTs are DNMT1, DNMT3A and DNMT3B. DNMT1 activity is crucial for the maintenance of DNA methylation, whereas DNMT3A and DNMT3B are de novo methyltransferases [29]. We adopted RT-PCR to detect the mRNA levels of DNMT1, DNMT3a and DNMT3b, the results showed that either daphnetin or 5-aza-dc can significantly reduce the mRNA levels of DNMT1, DNMT3a and DNMT3b in CIA rat synovial cells.

RA is a chronic autoimmune disease with characteristics of synovial inflammation. RASFs have been assigned a key role in the pathogenesis of RA and have been described as appearing transformed, tumor-like, or simply activated [30]. The mechanisms leading to hyperplastic synovitis are not fully understood, although paucity of apoptosis may contribute to the pathogenesis of RA. Apoptosis of RASFs may reduce secretion of a variety of inflammatory cell factors, which can decrease the intra-articular inflammation. So understanding how RASFs develop hyperplasia may be of great significance for clarifying the pathogenesis of RA and finding a new therapeutic target. This study used Annexin V/PI flow cytometry staining to observe the apoptotic condition of CIA rat synovial cells in each experimental group and found that daphnetin could significantly increase the apoptotic rate of CIA rat synovial cells. In addition, scanning and transmission electron microscopy observed the structural cell changes and found that CIA rat joint synovial cells in the daphnetin group presented a series of major apoptotic cell characteristics. All the results demonstrate that daphnetin has a pronounced effect on promoting CIA rat synovial cell apoptosis and increases the rate of apoptosis.

## Conclusion

Our findings demonstrate one of the molecular mechanisms behind daphnetin’s ability to promote CIA rat synovial cell apoptosis, which was through the inhibition of DNA methyltransferases DNMT1, DNMT3a and DNMT3b mRNA expression. Daphnetin reduces methylation and subsequently increase the expression of proapoptotic genes such as DR3, PDCD5, FasL and p53, thus promoting the apoptosis of the CIA rat synovial cells. This study provides a deeper insight into the development of daphnetin as a treatment of RA.

## Abbreviations

CIA: Collagen-induced arthritis
RA: Rheumatoid arthritis
RASFs: RA Synovial fibroblasts
DR3: Death receptor 3
PDCD5: Programmed death gene 5
DNMTs: DNA methyltransferases
MSP: Methylation specific PCR
SEM: Scanning electron microscopy
TEM: Transmission electron microscopy

## References

[1] Bartok B, Firestein GS (2010). Fibroblast-like synoviocytes: key effector cells in rheumatoid arthritis. Immunol Rev.

[2] Gao Q, Shan J, Di L, Jiang L, Xu H (2008). Therapeutic effects of daphnetin on adjuvant-induced arthritis rats. J Ethnopharmacol.

[3] Yao RF, Fu YY, Li S, Tu LN, Zeng XP, Kuang NZ (2011). Regulatory effect of daphnetin, a coumarin extracted from Daphne odora, on the balance of Treg and Th17 in collagen-induced arthritis. Eur J Pharmacol.

[4] Tu LN, Li S, Fu YY, Yao RF, Zhang ZQ, Yang SL, Zeng XP, Kuang NZ (2012). The therapeutic effects of daphnetin in collagen-induced arthritis involve its regulation of Th17 cells. Int Immunopharmacol.

[5] Park C, Kim GY, Jung JH, Kim WJ, Choi YH (2011). Pectenotoxin-2 induces G1 arrest of the cell cycle in synovial fibroblasts of patients with rheumatoid arthritis. Int J Mol Med.

[6] Adelheid K·H, Pavensta·Thomas P (2009). Cell death in rheumatoid arthritis [J]. Apoptosis.

[7] Luo SF, Fang RY, Hsieh HL, Chi PL, Lin CC, Hsiao LD, Wu CC, Wang JS, Yang CM (2010). Involvement of MAPKs and NF- kB in Tumor Necrosis Factor а-Induced Vascular Cell Adhesion Molecule 1 Expression in Human Rheumatoid Arthritis Synovial Fibroblasts. Arthritis & Rheumatism.

[8] Breton CV, Salam MT, Vora H, Gauderman WJ, Gilliland FD (2011). Genetic variation in the glutathione synthesis pathway, air pollution, and children’s lung function growth. Am J Respir Crit Care Med.

[9] Zhang H, Zhu Z, Meadows GG (2011). Chronic alcohol consumption decreases the percentage and number of NK cells in the peripheral lymph nodes and exacerbates B16BL6 melanoma metastasis into the draining lymph nodes. Cell Immunol.

[10] Baccarelli A, Wright RO, Bollati V, Tarantini L, Litonjua AA, Suh HH, Zanobetti A, Sparrow D, Vokonas PS, Schwartz J (2009). Rapid DNA methylation changes after exposure to traffic particles. Am J Respir Crit Care Med.

[11] Tarantini L, Bonzini M, Apostoli P, Pegoraro V, Bollati V, Marinelli B, Cantone L, Rizzo G, Hou L, Bertazzi P, Schwartz J, Baccarelli A (2009). Effects of particulate matter on genomic DNA methylation content and iNOS promoter methylation. Environ Health Perspect.

[12] Raychaudhuri S, Thomson BP, Remmers EF, Eyre S, Hinks A, Guiducci C, Catanese JJ, Xie G, Stahl EA, Chen R, Alfredsson L, Amos CI, Ardlie KG, Consortium BIRAC, Barton A, Bowes J, Burtt NP, Chang M, Coblyn J, Costenbader KH, Criswell LA, Crusius JB, Cui J, De Jager PL, Ding B, Emery P, Flynn E, Harrison P, Hocking LJ, Huizinga TW (2009). Genetic variants at CD28, PRDM1 and CD2/CD58 are associated with rheumatoid arthritis risk. Nat Genet.

[13] Stahl EA, Raychaudhuri S, Remmers EF, Xie G, Eyre S, Thomson BP, Li Y, Kurreeman FA, Zhernakova A, Hinks A, Guiducci C, Chen R, Alfredsson L, Amos CI, Ardlie KG, Consortium BIRAC, Barton A, Bowes J, Brouwer E, Burtt NP, Catanese JJ, Coblyn J, Coenen MJ, Costenbader KH, Criswell LA, Crusius JB, Cui J, de Bakker PI, De Jager PL, Ding B (2010). Genome-wide association study meta-analysis identifies seven new rheumatoid arthritis risk loci. Nat Genet.

[14] Tobon GJ, Youinou P, Saraux A (2010). The environment, geo-epidemiology, and autoimmune disease: Rheumatoid arthritis. J Autoimmun.

[15] Kim JK, Samaranayake M, Pradhan S (2009). Epigenetic mechanisms in mammals. Cell Mol Life Sci.

[16] Ballestar E (2011). Epigenetic alterations in autoimmune rheumatic diseases. Nat Rev Rheumatol.

[17] Meda F, Folci M, Baccarelli A, Selmi C (2011). The epigenetics of autoimmunity. Cellular & Molecular Immunology.

[18] Takami N, Osawa K, Miura Y, Komai K, Taniguchi M, Shiraishi M, Sato K, Iguchi T, Shiozawa K, Hashiramoto A, Shiozawa S (2006). Hypermethylated promoter region of DR3, the death receptor 3 gene, in rheumatoid arthritis synovial cells. Arthritis Rheum.

[19] Firestein GS, Budd RC, Harris T, Jr ED, McInnes IB, Ruddy S, Sergent JS (2009). Kelly’s textbook of rheumatology.

[20] Buchi F, Spinelli E, Masala E, Gozzini A, Sanna A, Bosi A, Ferrari G, Santini V (2012). Proteomic analysis identifies differentially expressed proteins in AML1/ETO acute myeloid leukemia cells treated with DNMT inhibitors azacitidine and decitabine. Leuk Res.

[21] Karouzakis E, Gay RE, Michel BA, Gay S, Neidhart M (2009). DNA hypomethylation in rheumatoid arthritis synovial fibroblasts. Arthritis Rheum.

[22] Meinecke I, Rutkauskaite E, Gay S, Pap T (2005). The role of synovial fibroblasts in mediating joint destruction in rheumatoid arthritis. Curr Pharm Des.

[23] Wang N, Lu HS, Guan ZP, Sun TZ, Chen YY, Ruan GR, Chen ZK, Jiang J, Bai CJ (2007). Involvement of PDCD5 in the regulation of apoptosis in fibroblast-like synoviocytes of rheumatoid arthritis. Apoptosis.

[24] Xingmin Z, Ming J, Dexian Z (1998). Rheumatoid arthritis synoviocyte hyperplasia and expression of fas and bcl-2 genes. Natl Med J China.

[25] Tang BX, You X, Zhao LD, Li Y, Zhang X, Tang FL, Ba DN, He W (2011). p53 in fibroblast-like synoviocytes can regulate T helper cell functions in patients with active rheumatoid arthritis. Chin Med J.

[26] Liu X, Tang K, Yu S, Wang Z, Su H (2012). Correlation between promoter methylation of p14 ARF, TMS1/ASC, and DAPK, and p53 mutation with prognosis in cholangiocar- cinoma. World Journal of Surgical Oncology.

[27] Okazaki R, Ootsuyama A, Yoshida Y, Norimura T (2011). Establishment of Methylation- Specific PCR for the Mouse p53 Gene. Molecular Biology International.

[28] Amaral CL, Lima Bueno RB, Burim RV, Queiroz RH, Bianchi Mde L, Antunes LM (2011). The effects of dietary supplementation of methionine on genomic stability and p53 gene promoter methylation in rat. Mutation Research/Genetic Toxicology and Environmental Mutagenesis.

[29] Turek-Plewa J, Jagodzinski PP (2005). The role of mammalian DNA methyltransferases in the regulation of gene expression. Cell Mol Biol Lett.

[30] Stanczyk J, Ospelt C, Gay RE, Gay S (2006). Synovial cell activation. Curt Opin Rheumatol.

